# Supplementary material for: Pan- and core- network analysis of co-expression genes in a model plant
Source: Sci Rep. 2016 Dec 16;6:38956. doi: 10.1038/srep38956 (PMC5159811; doi:10.1038/srep38956)
Supplement: Supplemental Files [file srep38956-s1.doc]

**Title**

Pan- and core- network analysis of co-expression genes in a model plant

**Running head**

Pan- and core- network analysis in Arabidopsis

**Authors**

Fei He^1*^, Sergei Maslov^1,2,3,4*^

^1^Biology Department, Brookhaven National Laboratory, Upton, NY 11973, USA.

^2^Department of Bioengineering,

^3^Carl R. Woese Institute for Genomic Biology,

^4^National Center for Supercomputing Applications, University of Illinois at Urbana-Champaign, Urbana, IL 61801, USA.

^*^To whom correspondence should be addressed ([maslov@illinois.edu](mailto:maslov@illinois.edu) for Sergei Maslov). Correspondence may also be Fei He ([plane83@gmail.com](mailto:plane83@gmail.com)).

**Supplemental Information**

Supplemental File 1. The detailed information for the subset of edges in the pan-network overlapping with the Protein-Protein Interaction network. The entire list of pan-network edges can be downloaded at the BitBucket (URL included in the file).

Supplemental File 2. Degrees and module memberships of all nodes in core- and pan-networks.

Supplemental File 3. The table of all edges of the core-network and their degree of universality (the number of networks they were observed).

Supplemental File 4. The enriched biological processes among network modules for pan- and core-networks.

Supplemental File 5. The detailed information for the gene AT1G55490 in the pan-network.

Supplemental File 6. The functional enrichment and preservation of gene modules identified in individual GEO dataset.

Supplemental File 7. The comprehensive list of publications reporting plant coexpression network meta-analysis along with the information on whether or not the study combines multiple datasets.

Supplemental File 8. Network similarity table measured by the fraction of shared edges (Eq. 2).

Supplemental File 9. Network similarity measured by the overlap between shared modules (Eq. 3).

Supplemental File 10. The table of GEO datasets used in this study.

Supplemental File 11. Our choice of parameters for the WGCNA software package including its ‘modulePreservation’ method.

Supplemental Figure S1. Histogram of PCC cutoffs used in 134 series-based network

Supplemental Figure S2. The difference between a more conventional approach where all expression samples are combined in a large table which is used to calculate a single “master” co-expression network and our pan-network approach formed by the union of all edges in co-expression networks based on expression series in individual experiments .

Supplemental Figure S3. PCC values for edges from core/pan-network within each of 134 experiments
