# Supplementary material for: Pan- and core- network analysis of co-expression genes in a model plant
Source: Sci Rep. 2016 Dec 16;6:38956. doi: 10.1038/srep38956 (PMC5159811; doi:10.1038/srep38956)
Supplement: Supplemental File 11 [file srep38956-s12.doc]

**Supplemental file 11. Our choice of parameters for the WGCNA software package including its ‘modulePreservation’ method.**

Parameters used when using WGCNA to detect modules in a dataset and evaluate preservation of modules in other datasets.

First, densely interconnected network modules were detected by Weighted Gene Co-Expression Network Analysis (WGCNA) software for each one of our 134 networks. Default parameters were used except the following:

*The smallest power between (2 and 20) which gives R^2^ above 0.8 was used.*

*A ‘signed’ adjacency and TOM matrix were constructed.*

*minModuleSize = 30*

*‘MEDissThres = 0.25*

Second, the method, ‘modulePreservation’ within WGCNA was utilized to calculate the preservation of each module in another dataset. Default parameters were used except the following:

*nPermutations = 100*

*randomSeed = 1*

*quickCor = 0*

*networkType = "signed"*
