# Supplementary material for: Pan- and core- network analysis of co-expression genes in a model plant
Source: Sci Rep. 2016 Dec 16;6:38956. doi: 10.1038/srep38956 (PMC5159811; doi:10.1038/srep38956)
Supplement: Supplementary Information [file srep38956-s13.pdf]

Figure S1

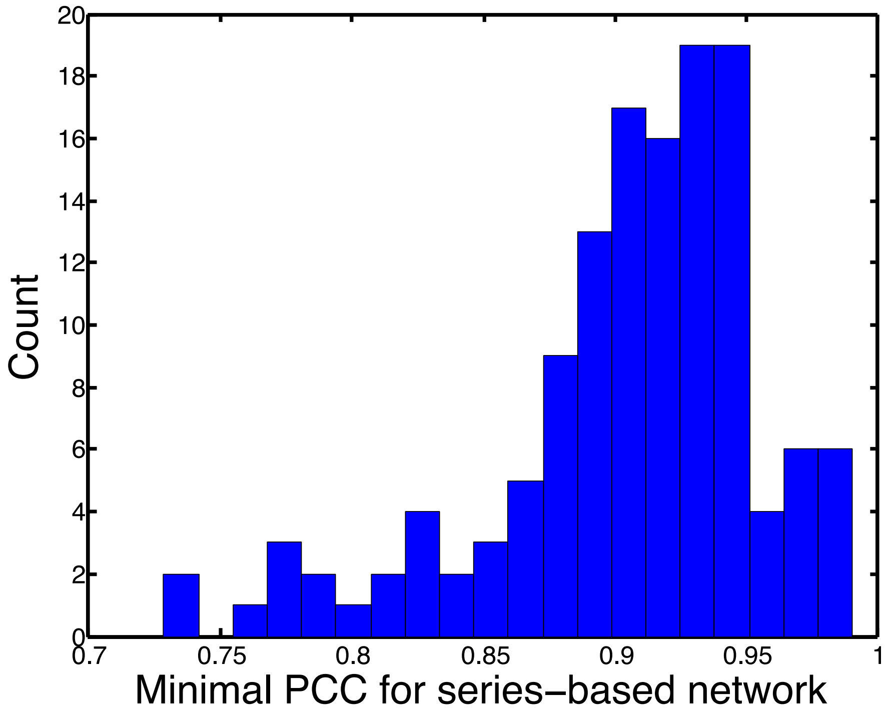

Figure S1. Histogram of minimal PCC values of edges within each series-based network.

Figure S2

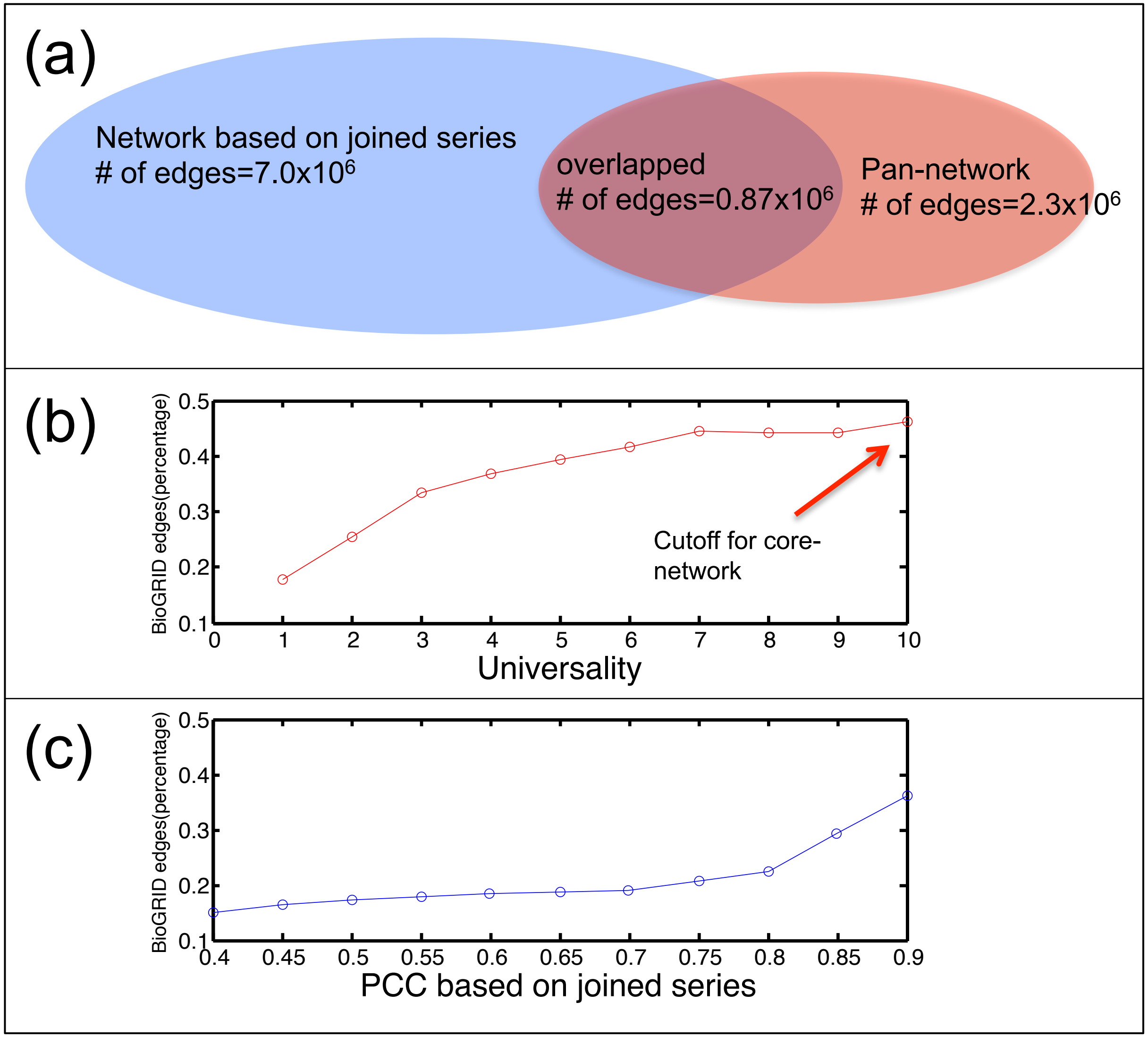

Figure S2. The difference between conventional methods and our approach.

(a) The blue area represents the size of the network inferred by calculating PCC across all 4410 microarray samples from 134 experiments. The cutoff is set as PCC=0.4. The red area represents the size of our pan network.

(b) The percentages of edges with experimental evidence are shown at different Universality.

(c) The percentages of edges with experimental evidence are shown at different PCC values of all 4410 samples.

Figure S3

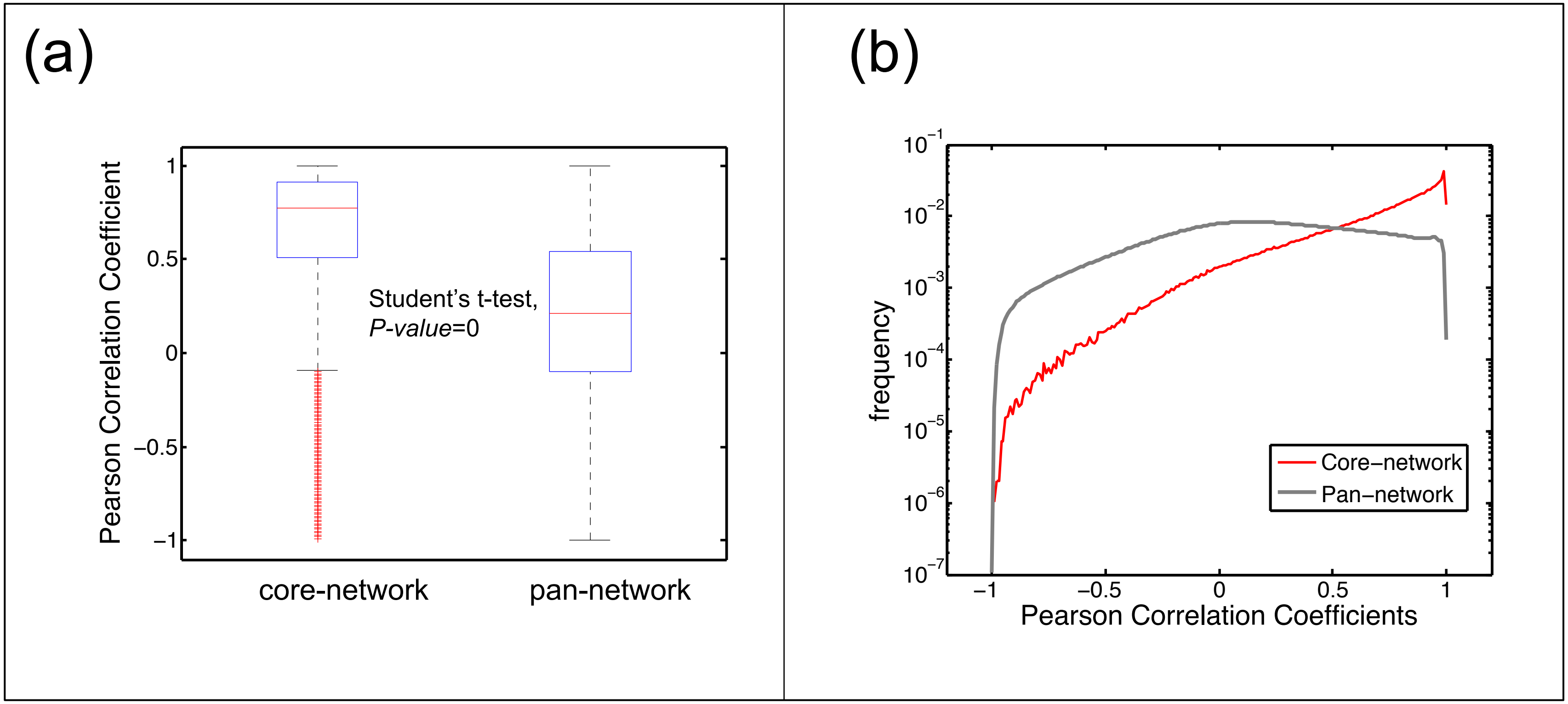

Figure S3. PCC values for edges from core/pan-network within each of experiment

(a) Box plot comparison of PCC values. Each PCC value represents the correlation strength for an edge of core/pan-network in one specific experiment.

(b) Distribution of PCC values in each of those 134 experiments.
